# Domain-selective thermal decomposition within supramolecular nanoribbons

**DOI:** 10.1038/s41467-021-27536-6

**Published:** 2021-12-20

**Authors:** Yukio Cho, Ty Christoff-Tempesta, Dae-Yoon Kim, Guillaume Lamour, Julia H. Ortony

**Affiliations:** 1 Department of Materials Science and Engineering, Massachusetts Institute of Technology, Cambridge, MA 02139 USA; 2 Functional Composite Materials Research Center, Korea Institute of Technology, Bondong, JB 55324 Korea; 3 LAMBE, Université Paris-Saclay, University of Evry, CNRS, Evry-Courcouronnes, France

**Keywords:** Molecular self-assembly, Synthesis and processing

## Abstract

Self-assembly of small molecules in water provides a powerful route to nanostructures with pristine molecular organization and small dimensions (<10 nm). Such assemblies represent emerging high surface area nanomaterials, customizable for biomedical and energy applications. However, to exploit self-assembly, the constituent molecules must be sufficiently amphiphilic and satisfy prescribed packing criteria, dramatically limiting the range of surface chemistries achievable. Here, we design supramolecular nanoribbons that contain: (1) inert and stable internal domains, and (2) sacrificial surface groups that are thermally labile, and we demonstrate complete thermal decomposition of the nanoribbon surfaces. After heating, the remainder of each constituent molecule is kinetically trapped, nanoribbon morphology and internal organization are maintained, and the nanoribbons are fully hydrophobic. This approach represents a pathway to form nanostructures that circumvent amphiphilicity and packing parameter constraints and generates structures that are not achievable by self-assembly alone, nor top-down approaches, broadening the utility of molecular nanomaterials for new targets.

## Abstract (web summary)

Molecular self-assembly in water is conventionally limited to amphiphilic molecules. This study harnesses sacrificial surface groups and a post-assembly chemical reaction to form nanostructures unachievable by spontaneous self-assembly alone.

## Introduction

Self-assembly of small molecules in water offers a bottom-up strategy to generate highly ordered nanostructures with dimensions on molecular (<10 nm) length scales^1–3^. The surface chemistries of the nanostructures can be tuned by modifying the hydrophilic head groups of the constituent amphiphiles. These assemblies exhibit high surface areas on the order of hundreds of m^2^/g^4^ and are therefore useful for applications that rely on interfacial interactions^5^. For example, the high surface areas, tunable surface chemistries, and high aspect-ratios of supramolecular nanofibers have led to advances in biomedical technologies^2,6,7^, including biological imaging^8^, tissue engineering^5,9^, drug delivery^10^, and biomineralization^11,12^, and newer applications such as water purification^13^, and photocatalysis^14^.

For small molecules to self-assemble in water, the overall molecular geometry must satisfy critical packing parameter constraints and, importantly, the head group of the constituent molecules must be sufficiently large and hydrophilic to counteract the hydrophobicity of the rest of the molecule^15^. The latter requirement precludes self-assembly of amphiphiles with sparingly soluble or insoluble head groups, even when their molecular geometries meet critical packing conditions^16,17^. However, many of these head group chemistries which do not lead to self-assembly would be desirable as nanostructure surface functionalities for target applications^13,18^. Extending self-assembly to small molecules that do not intrinsically possess sufficient amphiphilicity may allow us to dramatically diversify the surface chemistries of supramolecular nanostructures for a breadth of new applications.

Modifying small molecules in situ to induce nanostructure formation has been an area of active research in recent years. For instance, dynamic covalent chemistry has been used to trigger catalytic reactions between two species to form amphiphiles, causing energetically favorable assembly events^19–21^. Supramolecular amphiphiles formed by connecting two species via host–guest binding has been demonstrated as another route to self-assembly^22,23^. However, these strategies continue to rely on nanostructure surface chemistries with specific solvophilic interactions. In these—and nearly all conventional cases—molecular self-assembly represents the last step in the formation of nanostructures, likely because the structures tend to be intrinsically dynamic, causing them to rearrange, precipitate, or dissolve upon reaction.

Post-assembly modification of supramolecular assemblies may offer an alternative route to tailoring the nanomaterials’ surfaces for desired applications. In contrast to direct synthetic modifications of small molecule amphiphiles, only four methods of post-assembly treatments of supramolecular nanostructures have been reported in the literature: short peptides or drugs have been cleaved from nanofiber surfaces^24–26^, nanofibers containing diacetylene groups have been polymerized post-assembly^27^, photocatalysis has been demonstrated using perylene monoamide chromophore amphiphiles^28^, and peptide nanofibers have been functionalized by nanoparticles or proteins via native chemical ligation^29^. We surmise that post-assembly nanostructure modifications are uncommon because of the following: (1) orthogonal chemical reactivities must occur within each constituent molecule, giving rise to distinguishable “stable domains” and “reactive domains” after self-assembly; and (2) intermolecular interactions in the stable domain must be strong enough to prevent disassembly under the required reaction conditions.

Most self-assembly platforms developed for biomedical applications are not suitable for post-assembly reactions because their relatively weak internal interactions and dynamic mobility are important to their applications^30–33^. In a previous study, our group reported a self-assembling small molecule platform that incorporates aromatic amides (aramids) mimicking chemically inert Kevlar (poly(*p*-phenylene terephthalamide)) which could fulfill these requirements^4,34,35^. Aramid amphiphiles (AAs) exploit an extensive, collective hydrogen-bonding network to suppress dynamics once assembled. AAs spontaneously form planar nanoribbons in water with undetectable exchange dynamics and robust mechanical properties. The cohesion, strength, and high aspect-ratios of individual nanoribbons enable their processing to form flexible threads that are stable in both water and air^4^. Previous studies have demonstrated that the amide bonds in Kevlar are exceptionally stable upon heating because of their strong intramolecular cohesion^36–38^. In contrast, thermal decomposition of the head group domain is expected to occur at lower temperatures^39^. Therefore, we hypothesize that AAs have markedly different chemical stabilities between their ordered aramid structural domains and their hydrophilic head groups, which are highly hydrated and lack intermolecular stabilization.

**Fig. 1:**
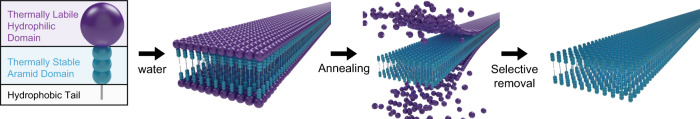
Schematic illustration of a domain-selective thermal decomposition pathway to construct hydrophobic supramolecular aramid nanoribbons. Aramid amphiphiles (AAs), composed of a thermally labile charged head group, an inert and stable aramid domain, and an aliphatic tail, are designed to spontaneously self-assemble in water into nanoribbons with suppressed exchange dynamics and superior mechanical properties. This design allows for selective removal of the hydrophilic nanoribbon surface by thermal annealing to create hydrophobic aramid nanoribbons post-assembly. Reproduced with permission^4^. Copyright 2021 Springer Nature.

In this work, we use self-assembly as an intermediate step to nanostructure formation by: (1) introducing non-reactive internal nanostructure domains that are resistant to molecular exchange and rearrangements, (2) incorporating sacrificial hydrophilic head groups to promote self-assembly in water, and (3) performing a post-assembly chemical reaction to remove the entire surface of a self-assembled nanoribbon via domain-selective decomposition. Specifically, we remove the hydrophilic surface of AA nanoribbons via thermal decomposition of sacrificial head groups and in turn create organized nanostructures with hydrophobic character (Fig. 1). This pathway to modify the surface chemistry of supramolecular structures by post-assembly domain-specific reactions greatly broadens the potential utility of molecular self-assembly, with new targets including superhydrophobic coatings and self-assembled molecular electronics^40–42^. Further, the demonstration of a sacrificial domain in a supramolecular nanostructure offers new opportunities in molecular nanostructure design and synthesis.

## Results

**Fig. 2:**
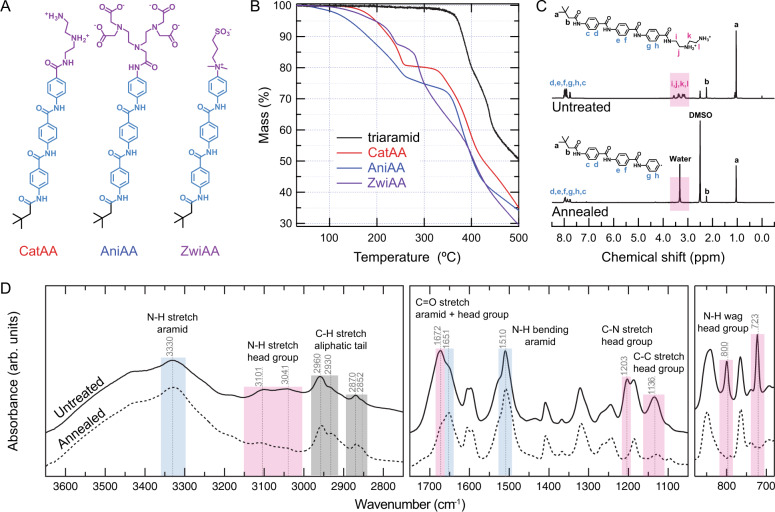
Heating induces selective cleavages of AA head groups. **A** CatAA, AniAA, and ZwiAA are AAs with cationic, anionic, and zwitterionic head groups, respectively (purple), an aramid structural domain (blue), and a short aliphatic tail (black). **B** Thermogravimetric analysis of CatAA, AniAA, and ZwiAA shows mass loss upon heating that first plateaus at approx. 250 °C. The triaramid control containing an analogous molecular structure as AAs but without a hydrophilic head group exhibits no mass loss in this range. **C** Representative ^1^H NMR analysis of AA (CatAA) demonstrates loss of the hydrophilic head group (pink area) with annealing. **D** FTIR spectra of CatAA before and after heating in air to 250 °C indicate that the head group (pink area) decomposes to leave the aramid (blue area) and alkyl tail (gray area) domains intact.

Thermogravimetric analysis with mass spectrometry (TGA-MS) was carried out to characterize the decomposition of AA nanoribbon head groups (Fig. 2A) upon heating. CatAA, AniAA, and ZwiAA—AAs with cationic, anionic, and zwitterionic head groups, respectively—exhibit a two-step degradation process, as shown in Fig. 2B. The first degradation step for each compound plateaus around 250 °C, with a mass loss proportional to the head group masses for CatAA and AniAA, and sulfur trioxide for ZwiAA (Fig. 2B). In contrast, a triaramid control compound with no head group (Supplementary Figs. 1–6) shows no decomposition upon heating until over 360 °C, confirming that the initial mass loss of AAs does not originate from decomposition of the hydrophobic domain. Decomposition onset temperatures corresponding to the thermally stable domain are similar in the range of 350–370 °C for CatAA, AniAA, and triaramid, whereas ZwiAA has an onset decomposition temperature of 280 °C (Supplementary Table 1). This instability is likely due to the positively charged quaternary amine adjacent to the aramid domain of ZwiAA^43^. Thus, we select CatAA and AniAA for further investigation due to the significantly disparate degradation onset temperatures between their hydrophobic and hydrophilic domains. Composition analysis of evolved gases from TGA further supports the decomposition of CatAA and AniAA into stable gases such as H_2_O, NH_3_, CO_2_, C_2_H_6_, and C_2_H_8_N_2_, which can be reconstructed back to the head group moieties (Supplementary Fig. 7). Multiple scans of TGA up to 250 °C reveal both CatAA and AniAA reach an equilibrium of mass loss at 250 °C (Supplementary Fig. 8) and do not decompose further by repeatedly heating under 250 °C (Supplementary Fig. 9).

Nuclear magnetic resonance (NMR) was used to identify the molecular structure of CatAA and AniAA before and after annealing at 250 °C. ^1^H NMR peaks between 3 and 4 p.p.m., corresponding to head group protons, are observed before but not after thermal treatment (Fig. 2C and Supplementary Fig. 10). In contrast, the chemical shifts of the aramid and aliphatic tail groups are maintained after heating. Similarly, ^13^C NMR peaks corresponding to head group carbons are not observed with annealing, whereas those from the aramid and aliphatic domains remain (Supplementary Figs. 11 and 12). Fourier-transform infrared spectroscopy (FTIR) spectra before and after annealing (Fig. 2D) are also consistent with NMR results. Absorbances corresponding to head group moieties in the FTIR spectra, labeled in pink, are not present after annealing, whereas those assigned to the aramid (blue) and aliphatic (black) tail domains are preserved. Further comparison of the spectra with triaramid and intermediate reactants before attaching head groups are shown in Supplementary Figs. 13 and 14. Finally, we observe the presence of the radical terminated triaramid product by MS after annealing both CatAA and AniAA (Supplementary Figs. 15). Based on the results of thermal and chemical characterization, we confirm that the annealed AAs terminate in a radical benzene after thermal cleavage, which follows previously reported thermal decomposition mechanisms for primary benzamides^39^. Therefore, having distinct thermal decomposition temperatures for different domains of the AA platform is confirmed, enabling selective cleavage of the head group. With the fastest head group decomposition kinetics to reach equilibrium at 250 °C, we select CatAA as the most suitable molecule for investigating the effects of selective thermal decomposition (Supplementary Fig. 8).

**Fig. 3:**
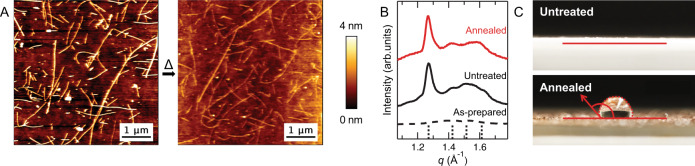
Site-selective thermal cleavage of AA hydrophilic head groups yields hydrophobic aramid nanoribbons. **A** Representative AFM of CatAA nanoribbons before (left) and after (right) annealing at 250 °C for 1 h in air. The nanoribbon morphology is maintained after annealing. **B** One-dimensional powder WAXS profiles of as-prepared CatAA before assembly (black dashed line), self-assembled CatAA nanoribbons after lyophilization (black solid line), and annealed aramid nanoribbons obtained from lyophilized CatAA nanoribbons (red solid line). Black dotted lines on the *x* axis are simulated peak positions of a unit cell with *a* = 8.30 Å, *b* = 4.95 Å, and *c* = 9.75 Å, and space group 26:*Pmc*2_1_ based on poly(*p*-benzamide). **C** Contact-angle measurements on films of as-cast CatAA nanoribbons and annealed nanoribbon films show a change from perfect wetting, with a contact angle of 0°, to low wettability, with a contact angle of 132°.

We investigated the ability of AA nanoribbons to retain their morphology and molecular organization throughout the thermal head group decomposition process. Atomic force microscope (AFM) and transmission electron microscope (TEM) images confirm that the nanoribbon morphology is maintained upon removal of the head groups (Fig. 3A and Supplementary Figs. 16 and 17). Further, preservation of long-range internal molecular organization of annealed aramid nanoribbons is confirmed by wide-angle X-ray scattering (WAXS). Peaks corresponding to the ordered aramid domain of annealed aramid nanoribbons are confirmed after annealing from lyophilized CatAA nanoribbons (Fig. 3B) and closely match a simulated poly(*p*-benzamide) unit cell as previously reported^4^. Variable temperature WAXS patterns of lyophilized CatAA and AniAA nanoribbons confirm the maintenance of characteristic hydrogen-bonding *d*-spacings throughout heating (Supplementary Fig. 18), which is observed as the strongest peak in Fig. 3B (*q* = 1.27 Å^−1^, *d* = 4.95 Å). Thus, AA nanoribbons maintain their nanoribbon morphology with well-defined internal order after thermal head group decomposition.

We hypothesize that the nanoribbon morphology and molecular packing imposed by the hydrophobic effect in solution persists after drying and heating to 250 °C due to deep kinetic traps originating from strong intermolecular interactions between aramid structural domains of neighboring AAs. Meanwhile, disassembly of CatAA nanoribbons after annealing can be triggered by introducing aprotic solvents such as dimethylformamide (DMF) and dimethyl sulfoxide (DMSO) (Supplementary Fig. 19). We confirmed that the peak corresponding to characteristic hydrogen bond *d*-spacings for the annealed nanoribbon is no longer observed after disassembly (Supplementary Fig. 20). Red shifts observed in ultraviolet-visible (UV-vis) spectra (Supplementary Fig. 21) further support the dissociation of hydrogen bonding in the aramid structural^44^.

The wettability of nanoribbon films before and after thermal removal of the head groups was probed by contact-angle measurements to confirm the transition from hydrophilic to hydrophobic surfaces. A film of CatAA nanoribbons is fully wetting (*θ* = 0°) when deposited and dried on a clean glass slide. After annealing, the film becomes hydrophobic, with a low-wetting contact angle of *θ* = 132° (Fig. 3C). In addition, a significant increase in turbidity and a color change of annealed aramid nanoribbons resuspended in water is observed (Supplementary Fig. 22), also indicating that CatAA nanoribbons become insoluble in water after heating. These results confirm that the removal of hydrophilic AA head groups is coupled with a drastic change in the nanoribbons surface-wetting properties.

**Fig. 4:**
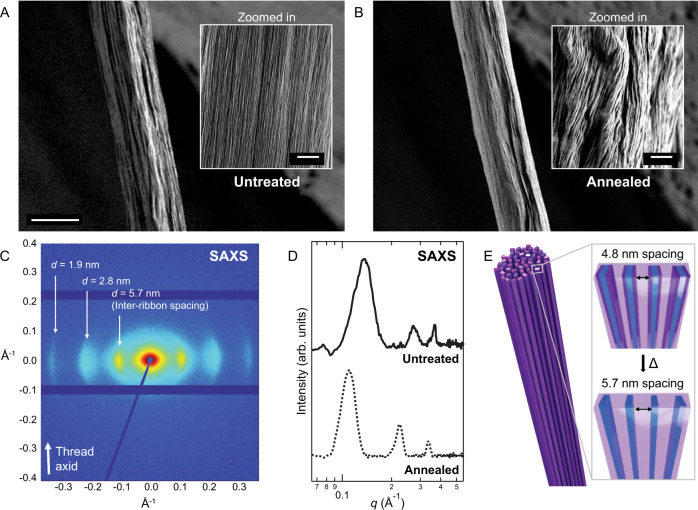
Nano- and microscale morphology, organized molecular packing, and long-range hierarchical order of AA nanoribbon threads are preserved after thermal head group decomposition. SEM of a 25 μm-diameter AA nanoribbon thread **A** before and **B** after annealing show long-range alignment of nanoribbon bundles (scale bar, 30 µm, inset scale bar, 2 µm). **C** Lamellar peaks in the SAXS pattern of annealed aramid nanoribbon threads indicate inter-ribbon spacing of 5.7 nm after annealing. **D** A 1-D SAXS scattering profile is obtained by integrating equatorial axes of **C**. The lamellar peaks corresponding to inter-ribbon spacing show a systematic increase in inter-ribbon spacing from 4.8 to 5.7 nm with heating, concomitant to a decrease of the nanoribbon width resulting from head group cleavage. **E** Schematic illustration of the increase of inter-ribbon distance from aligned semi-crystalline domains. Reproduced with permission^4^. Copyright 2021 Springer Nature.

Supramolecular nanoribbons that exhibit high surface charge have previously been shown to form gels upon addition of divalent counterions^31^ and are also capable of alignment into one-dimensional macroscopic aligned structures (“threads”)^4,45^. We explored the capability of AA nanoribbons to undergo domain-selective thermal decomposition after incorporation into aligned threads to determine whether their nanoscale morphology and hierarchical alignment are maintained. A macroscopic AA nanoribbon thread is shown in Fig. 4A and described elsewhere^4^, and annealed at 250 °C for 1 h to induce head group removal. Scanning electron microscopy (SEM) images of the annealed thread highlight preservation of macroscopic morphology and long-range alignment with local rearrangement after annealing (Fig. 4B). In addition, WAXS of the macroscopic threads show changes in unit cell dimensions from annealing, while maintaining analogous molecular packing as the previously reported untreated unit cell (Supplementary Fig. 23)^4^. In short, the unit cell parameters obtained from fitting the annealed thread exhibit a 9% contraction of the *d*-spacing along the hydrogen-bonding direction. These results indicate that packing in the direction of the nanoribbon long-axis becomes tighter as intermolecular electrostatic repulsion decreases with head group removal. At longer length scales, small-angle X-ray scattering (SAXS) (Fig. 4C) shows the preservation of uniform lamellar spacings between annealed aramid nanoribbons that are observed in untreated AA nanoribbon threads. A systematic shift of SAXS peaks in the equatorial direction reveals the inter-ribbon spacing increases from 4.8 to 5.7 nm with heating (Fig. 4D). The change in distance directly corresponds to the size of the cleaved CatAA head groups, leaving behind aligned hydrophobic nanoribbons with thicknesses of 3 nm after loss of the head group (Fig. 4E), whereas the untreated AA nanoribbons have a configuration of 4 nm thicknesses. Thus, we observe order is maintained in the molecular assembly on the scales of molecular packing, nanoribbon packing, and nanoribbon bundling to form macroscopic threads after annealing. In summary, post-assembly annealing of AA nanoribbons enabled us to produce high aspect-ratio and hydrophobic nanoribbons with pristine organization and homogeneous 3 nm thicknesses.

## Discussion

We have shown that orthogonal chemical stabilities within a single supramolecular nanostructure can be harnessed to support post-assembly chemical modifications. We demonstrated this capability using amphiphilic small molecules that spontaneously self-assemble to form nanoribbons in water and that contain a thermally stable aramid domain, i.e., AAs. AA nanoribbons exhibit strong intermolecular interactions that lead to chemical inertness in the aramid domain, whereas their hydrated and dynamic head groups are prone to decomposition upon heating. We showed by thermal and chemical characterization that decomposition of only the AA nanoribbon head group domain occurs upon heating, leaving behind a supramolecular nanoribbon containing entirely hydrophobic constituent molecules.

Contact-angle and X-ray scattering measurements confirmed that thermal decomposition of the surface groups leads to a hydrophilic-to-hydrophobic transition, whereas nanoribbon morphology and internal order are maintained. Further, we found that the nanoribbon surfaces may also be thermally removed after alignment of nanoribbons into a macroscopic one-dimensional thread configuration. Such pristine organization and homogeneous 3 nm thicknesses of supramolecular nanoribbons, both as individual structures and in condensed phases, are not achievable by top-down approaches.

We anticipate that, in addition to AAs, other self-assembly motifs containing both stable and reactive domains could also be of interest as reactive molecular nanostructures. This strategy may be exploited to generate supramolecular nanostructures that are composed of small molecules that do not naturally self-assemble or new supramolecular structures can be designed for post-assembly reactions by introduction of targeted reactive domains. Such a capability can extend the use of supramolecular chemistry to molecular structures outside of those that fulfill traditional amphiphilicity and critical packing parameter constraints, and in turn broaden the application space of supramolecular nanostructures.

## Methods

### General procedure

Synthesis details of CatAA, AniAA, and ZwiAA (1-(2-(4-(4-(4-(3,3-dimethylbutanamido)benzamido)benzamido)benzamido) ethyl)ethane-1,2-diaminium, 2,2′,2″,2‴-((((2-((4-(4-(4-(3,3-dimethylbutanamido) benzamido)benzamido)phenyl)amino)-2-ox-oethyl)azanediyl)bis(ethane-2,1-diyl))bis(azanetriyl))tetraacetate, and 3-((4-(4-(4-(3,3-dimethylbutanamido)benzamido)benzamido)phenyl) dimethylammonio)-propane-1-sulfonate, respectively), and “triaramid” (4-(3,3-dimethylbutanamido)-*N*-(4-(phenylcarbamoyl)phenyl)benzamide) are described in Supplementary Information (Supplementary Figs. 1–6). AA nanoribbon suspensions were prepared by dissolving AAs in deionized (DI) water and sonicating for 24 h in a bath sonicator. AA nanoribbons tested in a solid form are obtained by lyophilizing 2 wt% aqueous suspensions using a Labconco FreeZone benchtop freeze dryer. Shear alignment was performed by extruding CatAA nanoribbon suspensions into a 40 mM sodium sulfate (Na_2_SO_4_) aqueous solution. The AA threads were then pulled out of the solution and dried before further analysis. Annealing treatments were conducted at 250 °C for 1 h in air using a Fisher Scientific Isotemp oven.

### Thermal analysis

TGA-MS was carried out on a Discovery 55 (TA Instruments). Ten milligrams of powder was loaded for each experiment to measure mass loss with increasing temperature. Helium gas was used as the carrier gas for all experiments. The temperature was ramped from room temperature to 600 °C at a 5 °C/min scan rate. The composition of exhaust during heating was analyzed by a ThermoStar GSD 301 T3 mass spectrometer (Pfeiffer Vacuum).

### Chemical characterization

A Bruker Avance III DPX 400 was used for proton (^1^H) and carbon (^13^C) NMR. 20 mg of each sample were dissolved in 500 μL deuterated DMSO for analysis. FTIR was performed by dispersing the AA samples in KBr pellets and analyzing their absorbance on a ThermoFisher Scientific Nicolet 6700 and iS50. KBr-amphiphile pellets were prepared by mixing 0.1 mg of amphiphile powder with 0.5 g of KBr (Fisher Scientific, FTIR grade). A background spectrum of carbon dioxide-free air was subtracted from spectr, which were measured over the mid-IR range from 400 to 4000 cm^−1^. MS analysis was carried out to confirm the chemical composition of products and annealed samples. For matrix-assisted laser desorption/ionization (MALDI)–time-of-flight MS, a matrix solution was prepared by mixing excess (> 10 mg/mL) α-cyano-4-hydroxycinnamic acid in 500:500:1 acetonitrile:DI water:trifluoroacetic acid (by volume), vortexing for 1 min, centrifuging for 30 s, and retaining the supernatant. To produce a MALDI solution, 25 µL of this matrix solution, 25 µL of 1 mg/mL triaramid in DMF, and 1 µL of a 1 mg/mL solution of SpheriCal Peptide Low calibrant (as an internal standard) were mixed. MALDI spectra were captured on a Bruker Autoflex LRF Speed mass spectrometer. For direct analysis in real time (DART) MS, 1 mg/mL solutions of annealed amphiphile powders in dimethlyformamide were tested directly to obtain molecular weight spectra on a JEOL AccuTOF 4G LC-Plus equipped with an ionSense DART source. A poly(ethylene glycol) standard with a molecular weight of 415.25 g/mol was run in sequence with experimental samples for spectral calibration. The DART temperature was set at 400 °C and He was used as the working gas.

### Morphological characterization

A Bruker/JPK Nanowizard 4 AFM was used for capturing AFM images using OTESPA cantilevers (*k* = 80 N/m) in tapping mode in ambient air. A 2 wt% aqueous solution of CatAA was diluted 400 times (final concentration: 50 µg/ml) and deposited on clean Menzel-Glaser 18 × 18 mm rectangular glass coverslips. After 5 min of incubating the amphiphile solution on the glass, the surface was rinsed with DI water, dried with N_2_ gas, and used directly for AFM imaging. The same coverslip was heated in the oven at 250 °C for 65 min, cooled in ambient air to room temperature for 10 min, and then imaged to examine the annealed sample. An FEI Tecnai G2 Spirit TWIN microscope was used for capturing TEM images at an accelerating voltage of 120 kV. Grids were prepared by depositing 10 μL of a 1 mg/mL amphiphile suspension onto a continuous carbon grid (Electron Microscopy Sciences, 200 mesh, copper) for 20 s, blotting to remove the solution, depositing 10 μL of a 0.1% phosphotungstic acid solution onto the grid (Electron Microscopy Sciences), and blotting to remove the stain. UV-vis absorption spectra were captured by PerkinElmer LAMBDA 850+ UV/Vis spectrophotometer. A Zeiss MERLIN field-emission microscope was used to obtain SEM images at a 3 kV accelerating voltage and 200 pA probe current to resolve the higher-order structure of AA threads. SEM AA thread samples were coated with 10 nm Au (Quorum Q150T ES) to resolve local charging.

### Transmission X-ray scattering

WAXS and SAXS were performed on lyophilized AA nanoribbons and annealed aramid nanoribbons in 1.5 mm I.D. quartz capillary tubes (Hampton Research) and AA threads prepared on sample holders with a 6 mm hole. Measurements were performed on a SAXSLAB instrument using a Rigaku 002 microfocus X-ray source (CuK_α_ radiation, 1.5418 Å) and a DECTRIS PILATUS 300 K detector under high vacuum. WAXS and SAXS profiles were measured at a sample-to-detector distance of 109 and 459 mm, respectively, to capture the *q*-range from 0.02 to 2.38 Å^−1^.

### Contact-angle measurements

Samples for contact-angle measurements were prepared by drop casting and drying AA nanoribbon suspensions onto clean glass substrates. Contact angles of water were measured on an as-cast film and on an annealed film. Five microliters of DI water were pipetted onto each film and photographs of the droplets were taken and digitally analyzed for contact angle.

## Supplementary information

Supplementary Information

## Data Availability

The data generated and analyzed during this study are available from the corresponding author upon reasonable request.
