# Supplementary material for: Domain-selective thermal decomposition within supramolecular nanoribbons
Source: Nat Commun. 2021 Dec 20;12:7340. doi: 10.1038/s41467-021-27536-6 (PMC8688471; doi:10.1038/s41467-021-27536-6)
Supplement: Supplementary file 1 — Supplementary Information [file 41467_2021_27536_MOESM1_ESM.pdf]

Supplementary Information

Domain-selective thermal decomposition within supramolecular nanoribbons

Yukio Cho[1], Ty Christoff-Tempesta[1], Dae-Yoon Kim[2], Guillaume Lamour[3], Julia H. Ortony[1*]

[1]Department of Materials Science and Engineering, Massachusetts Institute of Technology, Cambridge, MA 02139, USA

[2]Functional Composite Materials Research Center, Korea Institute of Technology, Bondong, JB 55324, Korea

[3] LAMBE, Université Paris-Saclay, University of Evry, CNRS, Evry-Courcouronnes, France

*Correspondence to: ortony@mit.edu

This PDF file includes:

Supplementary Note 1: Synthesis .... Pg. 2

Supplementary Note 2: TGA-MS .... Pg. 8

Supplementary Note 3: NMR of decomposition product .... Pg. 10

Supplementary Note 4: FT-IR of decomposition product .... Pg. 13

Supplementary Note 5: DART-MS of decomposition product .... Pg. 15

Supplementary Note 6: TEM of annealed nanoribbon .... Pg. 16

Supplementary Note 7: Variable temperature WAXS .... Pg. 17

Supplementary Note 8: Disassembly of annealed nanoribbon .... Pg. 18

Supplementary Note 9: Solubility of decomposition product .... Pg. 20

Supplementary Note 10: WAXS of annealed nanoribbon threads .... Pg. 21

Supplementary Note 1: Synthesis

Materials and overview

Methyl 4-aminobenzoate (Sigma Aldrich, 98%), Boc-4-aminobenzoic acid (Chem Impex. 99%), 3,3-dimethylbutyric acid (Sigma Aldrich, 98%), *N,N*-dimethyl-*p*-phenylenediamine (DPP, Sigma Aldrich, 97%), *N*-Boc-*p*-phenylenediamine (BPP, Sigma Aldrich, 97%), 1,3-propanesultone (PPS, Sigma Aldrich, 99%), 1,4-bis-Boc-1,4,7-triazaheptane (BBT, Chem Impex, 100%), diethylenetriamine-*N,N,N'',N''*-tetra-*tert*-butyl acetate-*N'*-acetic acid (DPTA, Combi Blocks, 95%), aniline (Sigma Aldrich, 99.5%), 1-ethyl-3-(3-dimethylaminopropyl)carbodiimide hydrochloride (EDC, TCI Chemicals, 98%), 4-dimethylaminopyridine (DMAP, TCI Chemicals, 99%), 1-hydroxybenzotriazole hydrate (HOBt, TCI Chemicals, 97%), N,N- Diisopropylethylamine (DIPEA, Alfa Aesar, 99%), lithium hydroxide monohydrate (LiOH.H2O, Alfa Aesar, 98%), sodium bicarbonate ($NaHCO_3$, Alfa Aesar, 99%), hydrochloric acid (HCl, Alfa Aesar, 36%), sodium sulfate (Na_2SO_4, Fisher Scientific, 99%), and trifluoroacetic acid (TFA, Alfa Aesar, 99%) were used as received without further purification.

This study relied on carbodiimide-mediated coupling reactions, deprotection of *tert*-butyloxycarbonyl (Boc) groups, and ester hydrolysis to synthesize aramid amphiphile and control compounds. The head group of ZwiAA was realized by quaternization of a tertiary amine with propanesultone. The chemical composition of intermediates and products were confirmed thorugh ^{1}H and ^{13}C nuclear magnetic resonance (NMR) and mass spectroscopy. The synthesis and NMR spectra of CatAA, AniAA, ZwiAA, and their intermediates are reported previously[1], and the NMR spectra of the new compound in this study (triaramid) is given in Supplementary Figure 5 and 6. Synthesis schemes and details on each of compounds CatAA, AniAA, ZwiAA, and triaramid, and their intermediates are given below.

CatAA

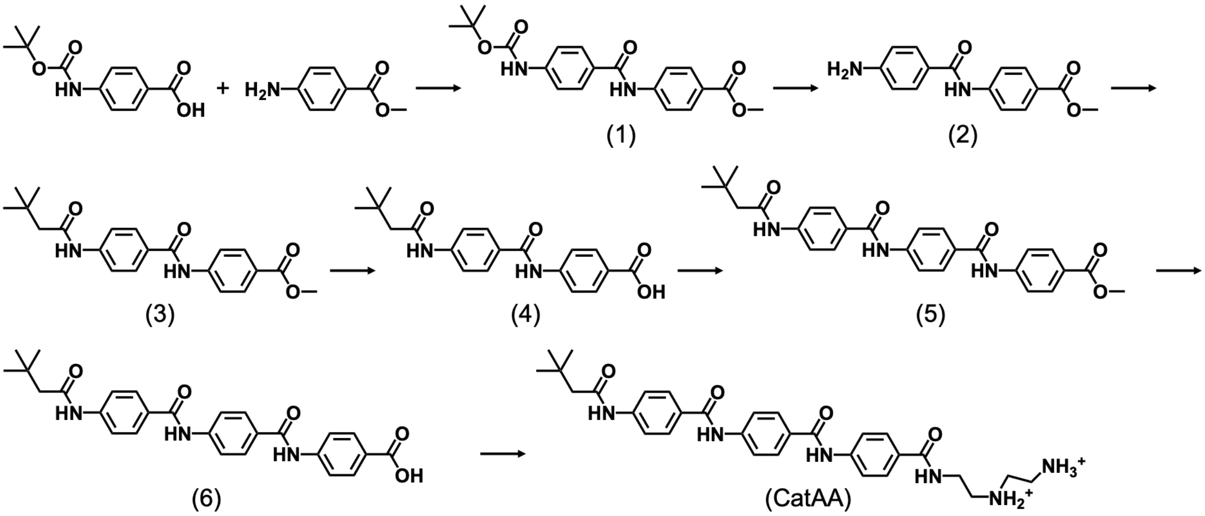

Supplementary Figure 1 | Synthesis scheme to obtain the CatAA amphiphile

Methyl 4-(4-((*tert*-butoxycarbonyl)amino)benzamido)benzoate (1): Boc-4-aminobenzoic acid (25 mmol), methyl 4-aminobenzoate (25 mmol), EDC (30mmol), and HOBt (30mmol) in

chloroform (200 mL) was stirred for 24 h at r.t. The solvent was then removed *in vacuo* and the product was sonicated overnight. The product was obtained by precipitation in deionized water, filtration, and washing with excess methanol (yield: 37%).

Methyl 4-(4-aminobenzamido)benzoate (2): TFA (25 ml) was mixed with compound 1 (15 mmol) in chloroform (150 mL). After stirring for 24 h at r.t., volatile components were removed *in vacuo* and the product was precipitated in saturated $NaHCO_3$ aqueous solution. The product was obtained by filtration and dried under vacuum (yield: 99%).

Methyl 4-(4-(3,3-dimethylbutanamido)benzamido)benzoate (3): EDC (45 mmol), DMAP (45 mmol), compound 2 (15 mmol), and 3,3-dimethylbutyric acid (30 mmol) were mixed in dimethylformamide (150 mL) for 24 h at 60 °C. After the reaction, aqueous brine (210 g NaCl/L, 500 mL) was added to the solution to precipitate the crude product, which was collected by filtration. The crude product was further washed with methanol and dried under vacuum to obtain the final product (yield: 90%).

4-(4-(3,3-dimethylbutanamido)benzamido)benzoic acid (4): $LiOH \cdot H_2O$ (150 mmol) in deionized water (60 mL) was mixed with compound 3 (15 mmol) in tetrahydrofuran (240 mL) and methanol (120 mL). The solution was refluxed for 24 h, and then neutralized with 1% aqueous HCl. The precipitate was obtained by filtration, washed with deionized water, and dried under vacuum to realize the product (yield: 98%).

Methyl 4-(4-(4-(3,3-dimethylbutanamido)benzamido)benzamido)benzoate (5): EDC (30 mmol), DMAP (30 mmol), compound 4 (10 mmol), and methyl 4-aminobenzoate (30 mmol) were mixed in dimethylformamide (150 mL) for 24 h at 50 °C. After the reaction, aqueous brine (210g/L, 750 mL) was added to the solution to precipitate the crude product, which was collected by filtration. The crude product was further washed with methanol and dried under vacuum to obtain the final product (yield: 85%).

4-(4-(4-(3,3-dimethylbutanamido)benzamido)benzamido)benzoic acid (6): LiOH·H2O (50 mmol) in deionized water (20 mL) was mixed with compound 5 (5 mmol) in tetrahydrofuran (80 mL) and methanol (40 mL). The solution was refluxed for 24 h and then neutralized with 1% aqueous HCl. The precipitate was obtained by filtration, washed with deionized water, and dried under vacuum to obtain the final product (yield: 98%).

1-(2-(4-(4-(4-(3,3-dimethylbutanamido)benzamido)benzamido)benzamido)ethyl)ethane-1,2-diaminium (CatAA): Compound 6 (0.8 mmol), BBT (1.6 mmol), EDC (2.4 mmol), HOBt (2.4 mmol), and DIPEA (2.4 mmol) were mixed in dimethylformamide (30 mL) and dichloromethane (30 ml) at room temperature for 24 h. After the reaction, the solvent was removed *in vacuo* and the remaining residue was washed with deionized water and cold acetonitrile several times. The isolated compound was then mixed with TFA (8 mL) in methylene chloride (80 mL) for 24 h. The volatile fraction was removed *in vacuo*, and diethyl ether was added to precipitate the product, which was collected by filtration (yield: 80%).

AniAA

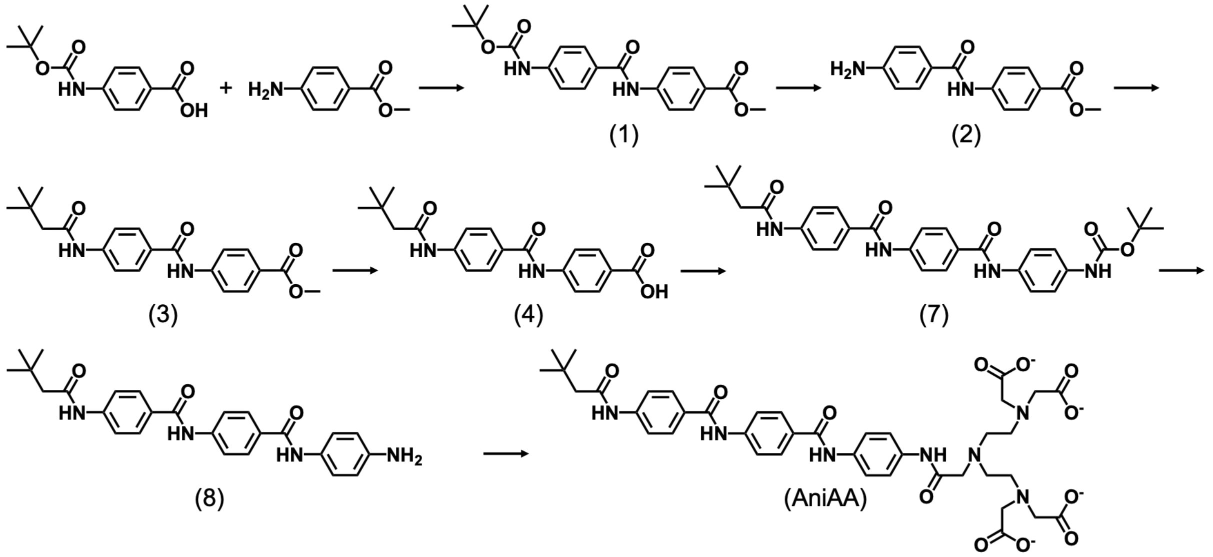

Supplementary Figure 2 | Synthesis scheme to obtain the AniAA amphiphile

tert-Butyl 4-(4-(4-(3,3-dimethylbutanamido)benzamido)benzamido)phenylcarbamate (7): Compound 4 (0.85 mmol), BPP (2.55 mmol), EDC (2.55 mmol), and DMAP (2.55 mmol) were mixed in dimethylformamide (20 mL) at room temperature for 24 h. The solvent was then removed *in vacuo* and the crude mixture was mixed with water to obtain a precipitate, which was obtained by filteration and washed with methanol to yield the final product (yield: 81%).

N-(4-(amino)phenyl)-4-(4-(3,3-dimethylbutanamido)benzamido)benzamide (8): TFA (500 μL) and compound 7 (0.55 mmol) were mixed in methylene chloride (15 mL) for 6 h at room temperature. Then, the volatile components were removed *in vacuo* and the remaining mixture was washed with saturated $NaHCO_3$ solution to obtain the final product, which was obtained by filtration and dried under vacuum (yield: 99%).

2,2',2'',2'''-((((2-((4-(4-(4-(3,3-dimethylbutanamido)benzamido)benzamido)phenyl)amino)-2-ox-oethyl)azanediyl)bis(ethane-2,1-diyl))bis(azanetriyl))tetraacetate (AniAA): Compound 8 (0.29 mmol), DPTA (0.58 mmol), EDC (1.17 mmol), and DMAP (1.17 mmol) were mixed in dimethylformamide (20 mL) at 50 °C for 72 h. After the reaction, the solvent was removed *in vacuo*. The resulting residue was purified by silica gel flash column chromatography with a 7:1 mixture of tetrahydrofuran : chloroform by volume as the eluent. The isolated compound was mixed with TFA (500 μL) in methylene chloride (15 mL) for 48 h. Volatile components were removed *in vacuo* and tetrahydrofuran was added to suspend the product, which was obtained by filtration (yield: 67%).

ZwiAA

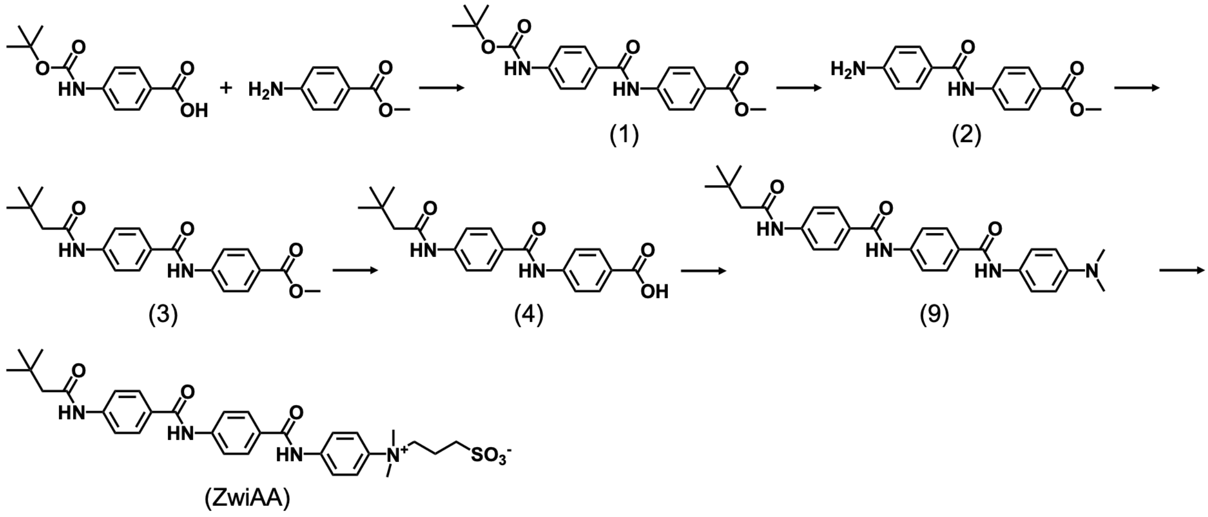

Supplementary Figure 3 | Synthesis scheme to obtain the ZwiAA amphiphile

N-(4-(dimethylamino)phenyl)-4-(4-(3,3-dimethylbutanamido)benzamido)benzamide (9): Compound 4 (0.85 mmol), DPP (2.55 mmol), EDC (2.55 mmol), and HOBt (2.55 mmol) were mixed in dimethylformamide (20 mL) at 50 °C for 24 h. After the reaction, the solvent was removed *in vacuo* and the remaining residue was precipitated in water and obtained by filtration. The filtrate was washed with chloroform several times to obtain the final product (yield: 78%).

3-((4-(4-(4-(3,3-dimethylbutanamido)benzamido)benzamido)phenyl)dimethylammonio) propane-1-sulfonate (ZwiAA): PPS (5 mL) was slowly injected into a solution of compound 9 (1.85 mmol) dissolved in dimethylformamide (15 mL) and tetrahydrofuran (15 mL), and the solution was then stirred for 48 h in a sealed pressure tube at 70 °C. The volatile fraction was removed *in vacuo* and a precipitate was obtained by suspension in acetonitrile (50 mL). The precipitate was filtered and dried under vacuum to obtain the final product (yield: 85%).

Triaramid

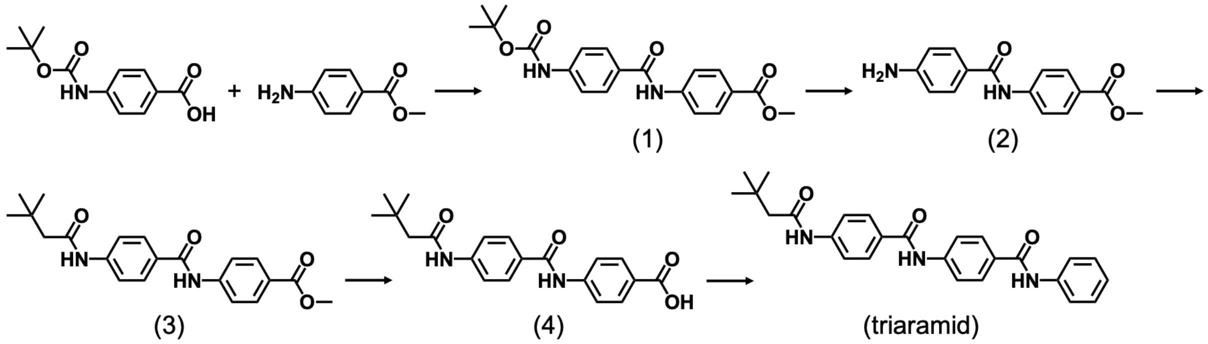

Supplementary Figure 4 | Synthesis scheme to obtain the triaramid control compound.

4-(3,3-dimethylbutanamido)-*N*-(4-(phenylcarbamoyl)phenyl)benzamide (triaramid control) was synthesized by adding aniline (1.27 mmol), 1-ethyl-3-(3-dimethylaminopropyl)carbodiimide hydrochloride (1.27 mmol) and 4-dimethylaminopyridine (1.27 mmol) to a solution of compound 4 in dimethylformamide (15 mL). The solution was

stirred for 20 h at 60 °C. After the reaction, the solvent was removed *in vacuo*, and the product was precipitated with aqueous brine (5 wt% sodium chloride in deionized water), collected by filtration, and washed with excess methanol to obtain the final product. ^{1}H NMR (400 MHz, DMSO-d_6): δ = 7.97 (m, 6H), 7.78 (m, 4H), 7.36 (t, 2H), 7.10 (t, 1H), 2.25 (s, 2H), 1.05 (s, 9H) ppm. ^{13}C NMR (400 MHz, DMSO-d_6): δ = 171.1, 165.6, 165.4, 142.9, 139.8, 129.9, 129.1, 124.0, 120.8, 119.8, 118.7, 50.1, 31.4, 30.1 ppm. MS (MALDI-ToF) $[M + Na]^+$ m/z calculated 452.20; $[M + Na]^+$ found 452.19.

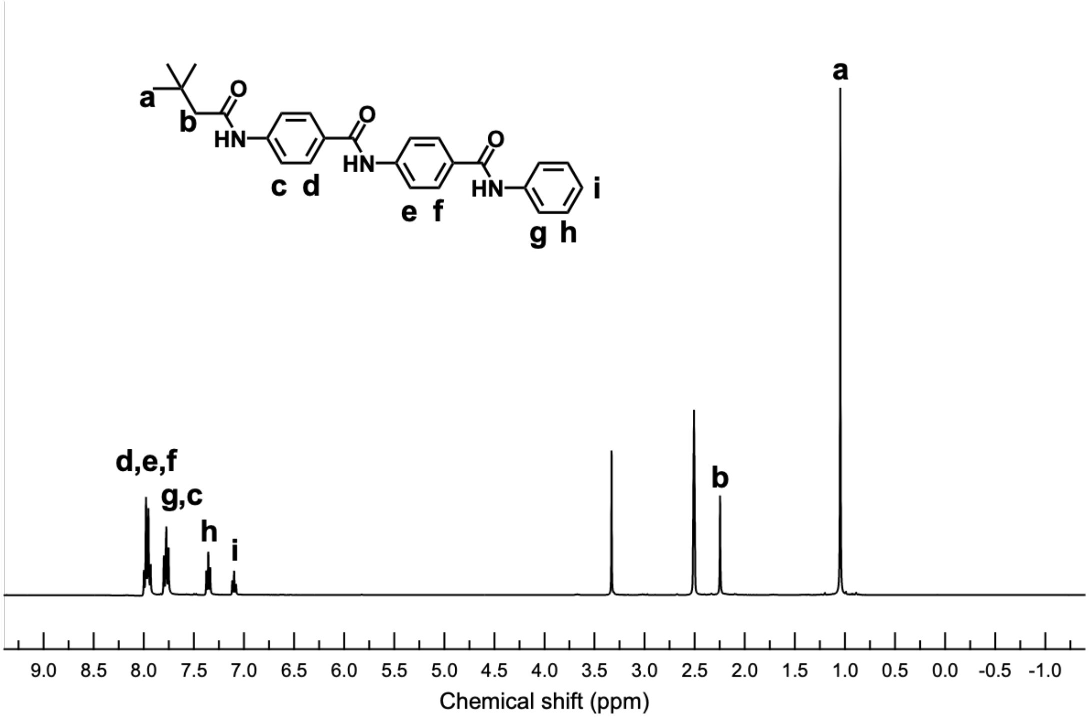

Supplementary Figure 5 | ^{1}H NMR spectra of the triaramid control compound.

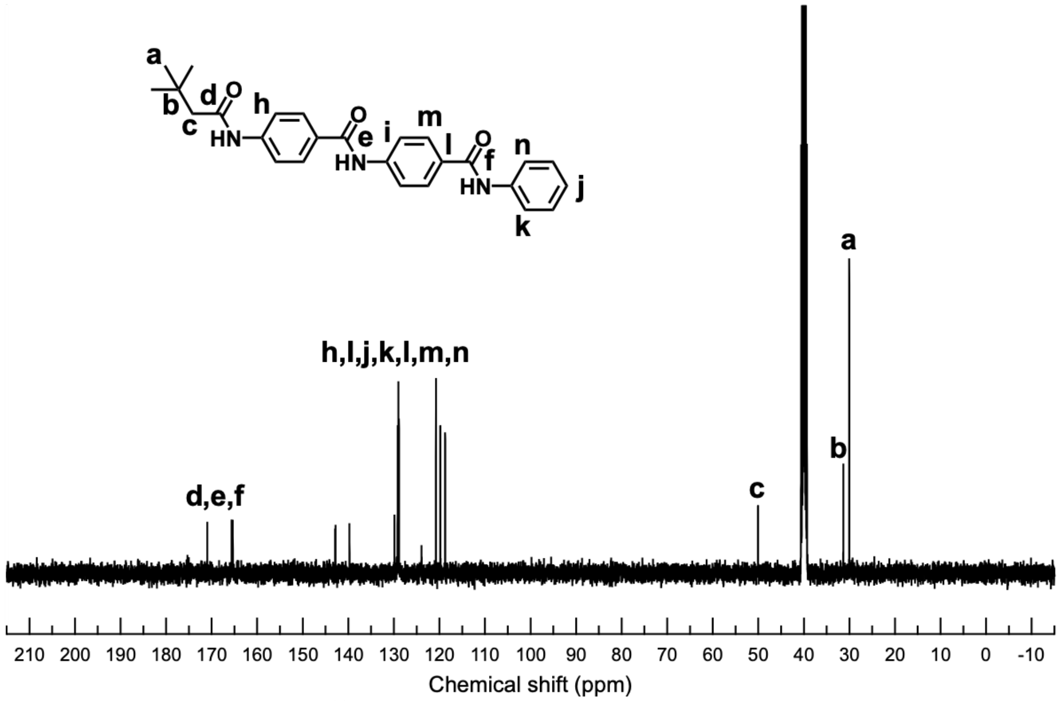

Supplementary Figure 6 | ^{13}C NMR spectra of the triaramid control compound.

Supplementary Note 2: TGA-MS

Supplementary Table 1 | Decomposition onset temperature extrapolated from TGA curves.

AAs	1st onset temperature	2nd onset temperature
**CatAA**	208.5 °C	352.3 °C
**AniAA**	216.9 °C	358.2 °C
**ZwiAA**	199.1 °C	279.2 °C
**triaramid**	367.6 °C	------

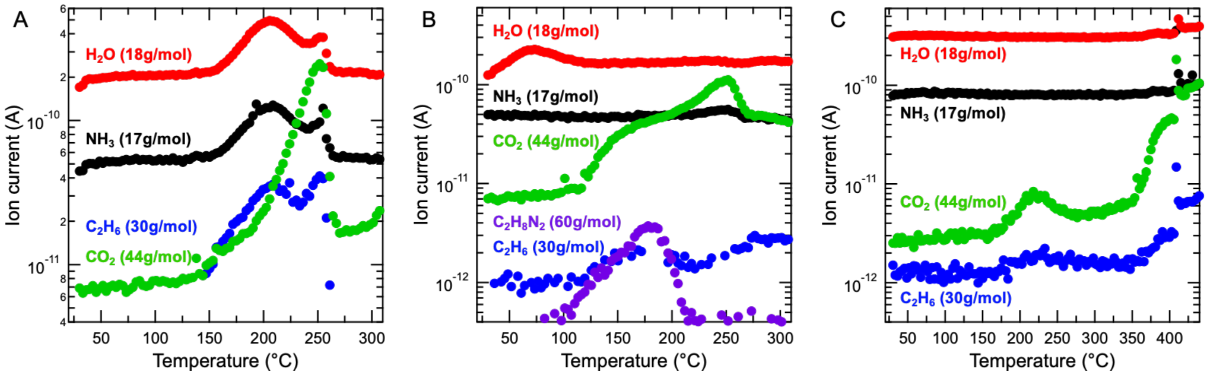

Supplementary Figure 7 | Head group decomposition products detected by mass spectrometry upon heating during thermogravimetric analysis of (**A**) CatAA, (**B**) AniAA, and (**C**) the triaramid compound. Masses corresponding to H_2O, NH_3, CO_2, C_2H_6, and $C_2H_8N_2$, are all observed as head group decomposition products between rt and 250 °C in CatAA and AniAA (Figure S3A and S3B). No decomposition products are detected in the evolved gas from the triaramid sample except adsorbed CO_2 at 180°C as shown in Figure S3C. These results support that the head groups of aramid amphiphiles are selectively decomposed during heating.

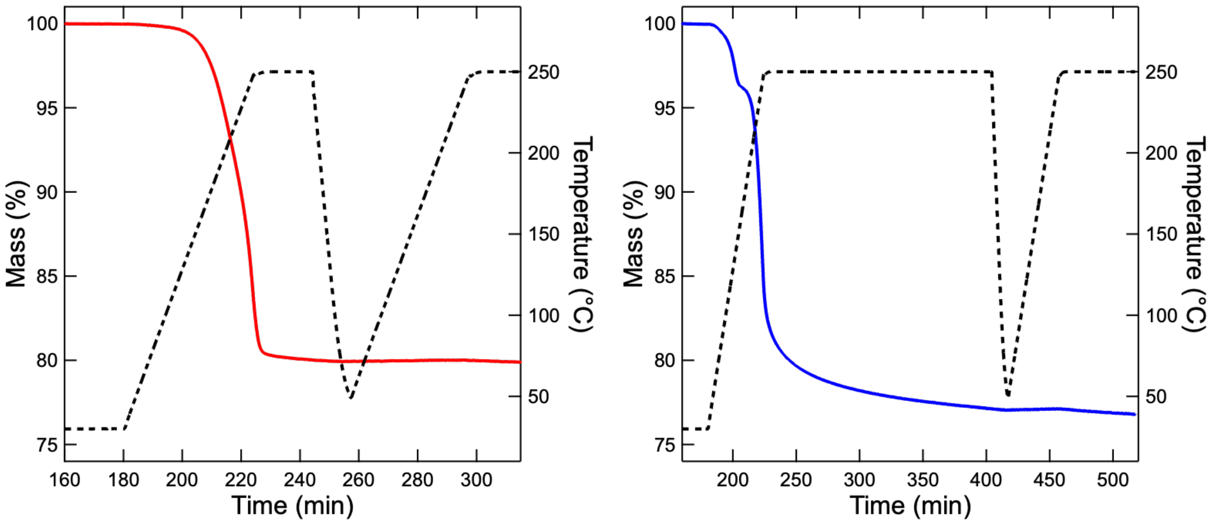

Supplementary Figure 8 | Thermogravimetric analysis of (**A**) CatAA and (**B**) AniAA upon heating to 250°C in Cycle 1, maintaining for 1-3h, cooling the sample to rt, and heating the sample again to 250°C in Cycle 2. Red and blue solid lines represent mass loss with heating, and the black dotted line represents temperature as a function of time. Both CatAA and AniAA reach equilibrium mass losses at 250 °C. The first 180 minutes, not shown, are an isothermal step at 30 °C for initial equilibrium.

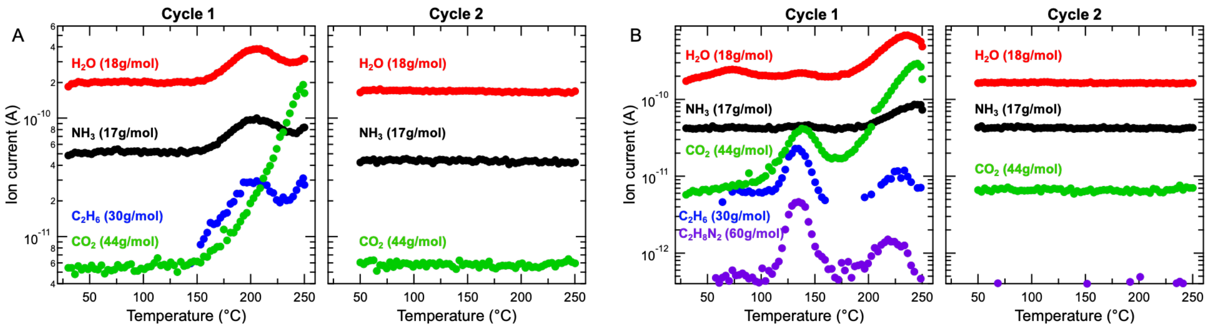

Supplementary Figure 9 | Head group decomposition products detected by mass spectrometry upon heating to 250°C in Cycle 1, cooling the sample to rt, and heating the sample again to 250°C in Cycle 2 during thermogravimetric analysis of (**A**) CatAA and (**B**) AniAA. No decomposition is detected in this second cycle, indicating molecular decomposition is selective up to 250°C and is not a continuous process. Signal of C_2H_6 (blue dots) and $C_2H_8N_2$ (purple dots) are under the detection limit for Cycle 2.

Supplementary Note 3: NMR of decomposition product

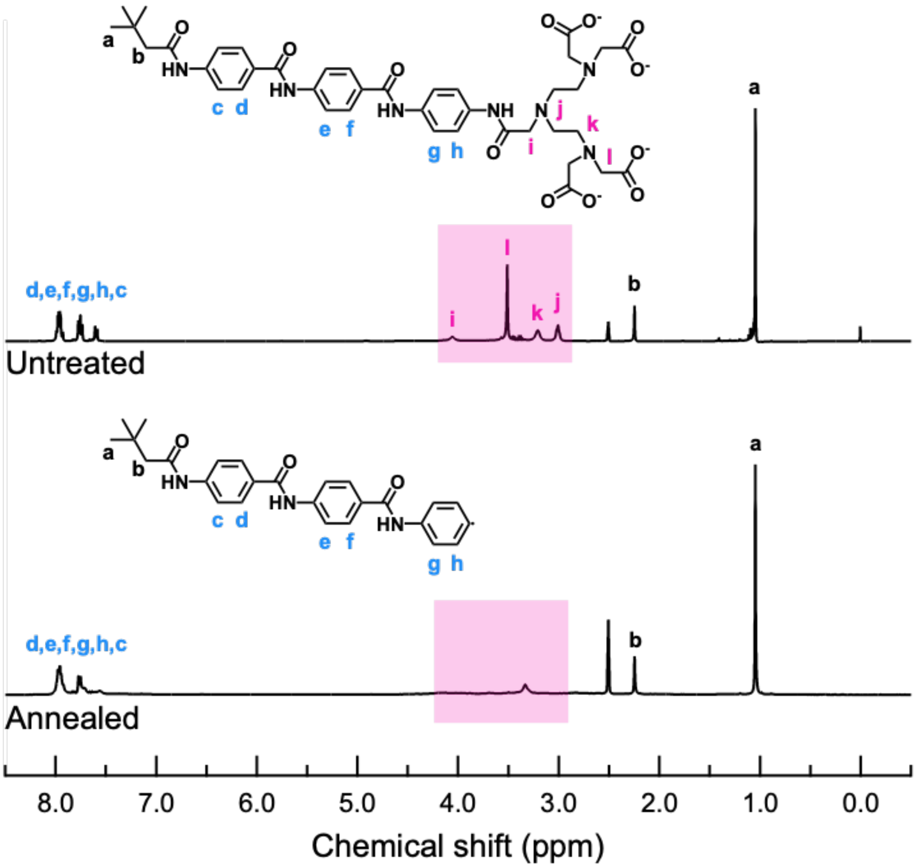

Supplementary Figure 10 | ^{1}H NMR analysis of AniAA before and after heating in air to 250°C indicate that the head group decomposes to leave the aramid and alkyl tail domains intact. Untreated adapted from supplementary reference 1, Copyright 2021 Springer Nature

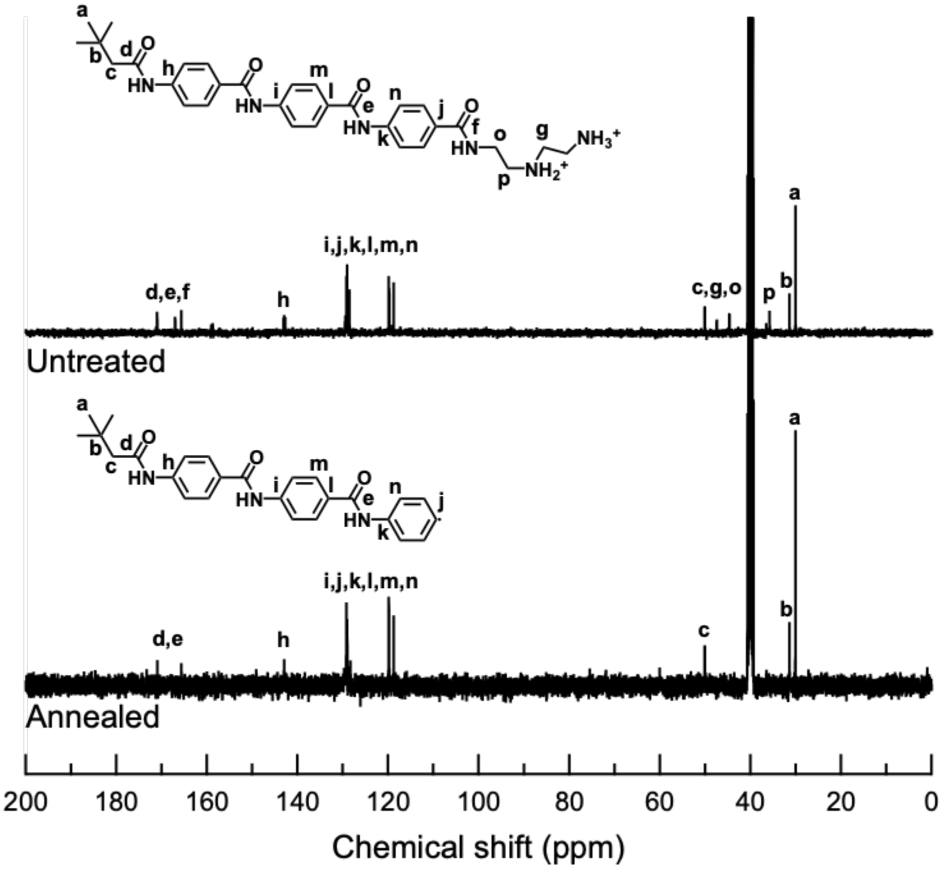

Supplementary Figure 11 | ^{13}C NMR analysis of CatAA before and after heating in air to 250°C indicate that the head group decomposes to leave the aramid and alkyl tail domains intact. Untreated adapted from supplementary reference 1, Copyright 2021 Springer Nature

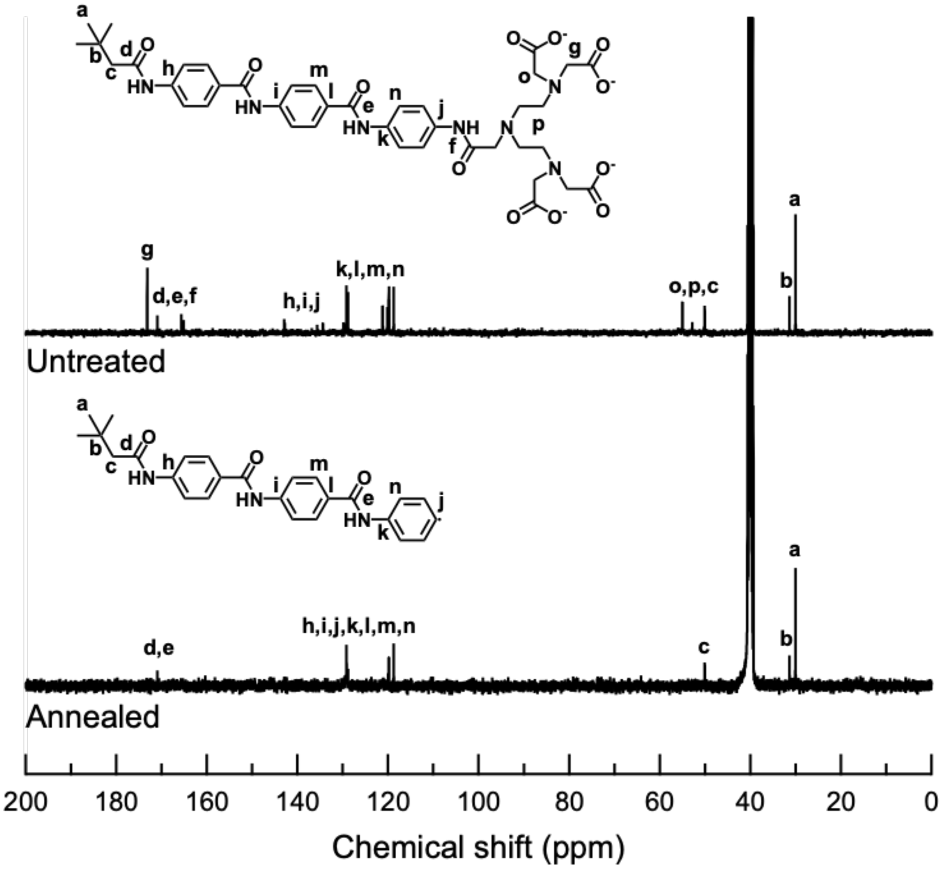

Supplementary Figure 12 | ^{13}C NMR analysis of AniAA before and after heating in air to 250°C indicate that the head group decomposes to leave the aramid and alkyl tail domains intact. Untreated adapted from supplementary reference 1, Copyright 2021 Springer Nature

Supplementary Note 4: FT-IR of decomposition product

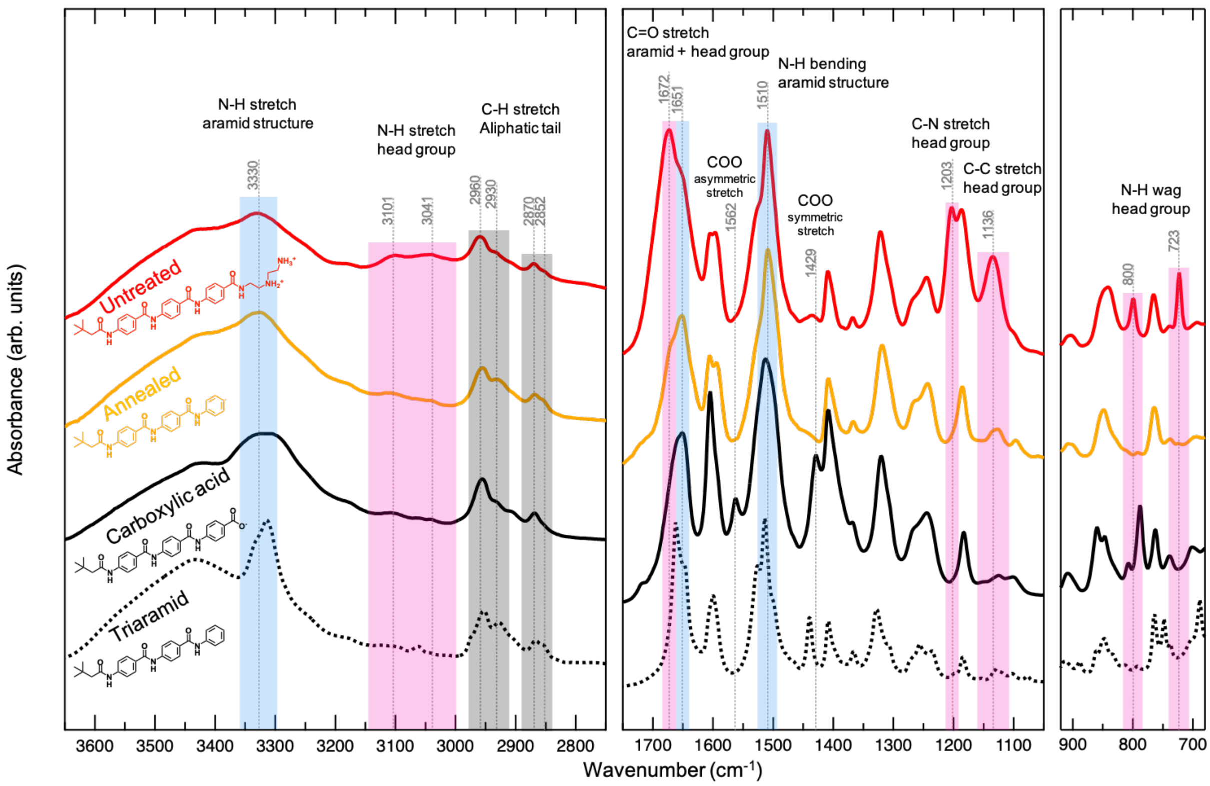

Supplementary Figure 13 | FTIR spectra of CatAA before and after heating in air to 250°C, an intermediate compound with a carboxylic acid terminating group as previously synthesized[1], and the triaramid. Pink, blue and gray regions correspond to head group, aramid structural domain, and aliphatic tail regions of CatAA, respectively. N-H (3350-3300 and 1520-1500 cm^{-1}), C=O (1670-1640 cm^{-1}), and C-H (2980-2850 cm^{-1}) stretching or bending peaks correspond to aramid structural domain and aliphatic tails and are observed for all compounds[2,3]. Specific N-H (3120-3080, 3060-3030, 800 and 723 cm^{-1}), C=O (1680-1660 cm^{-1}), C-N (1203 cm^{-1}) and C-C (1150-1120 cm^{-1}) stretching or wagging peaks corresponding to the cationic head group were only observed in unheated CatAA[4,5]. Furthermore, the ester moiety between the aramid structural domain and head group (-COO-) was confirmed to cleave after annealing due to the loss of characteristic -COO- symmetric and asymmetric stretches. Hence, the FTIR spectra are consistent with selective head group decomposition. Slightly differing fingerprints between annealed CatAA and triaramid in the aromatic C-H bending region (900-700 cm^{-1}) suggest a subtle chemical difference between them. This difference likely arises from trapping of evolved head groups in the local environment during annealing.

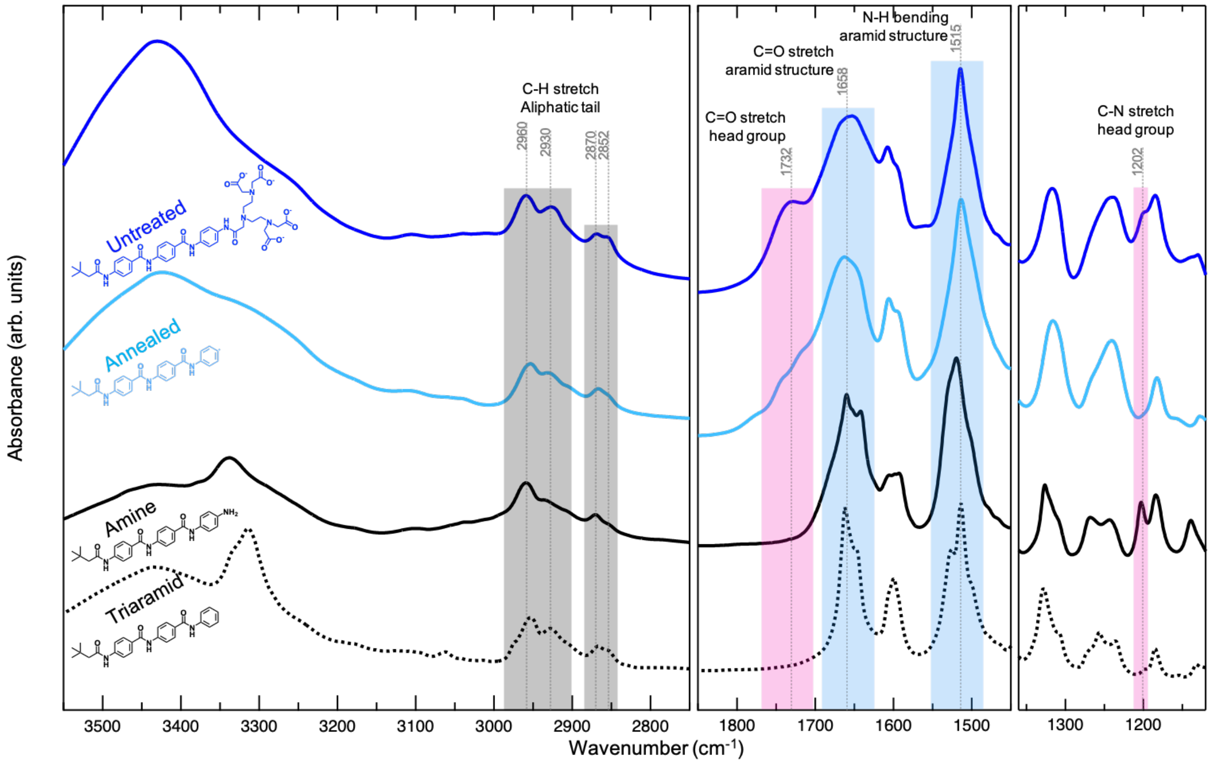

Supplementary Figure 14 | FTIR spectra of AniAA before and after heating in air to 250°C, an intermediate compound with an amine terminating group as previously synthesized[1], and the triaramid control. Pink, blue and gray regions correspond to head group, aramid structural domain, and aliphatic tail regions of AniAA, respectively. Specific C=O (1740-1720 cm^{-1}) and C-N (1202 cm^{-1}) stretching peaks corresponding to the anionic head group were only observed in unheated AniAA[6].

Supplementary Note 5: DART-MS of decomposition product

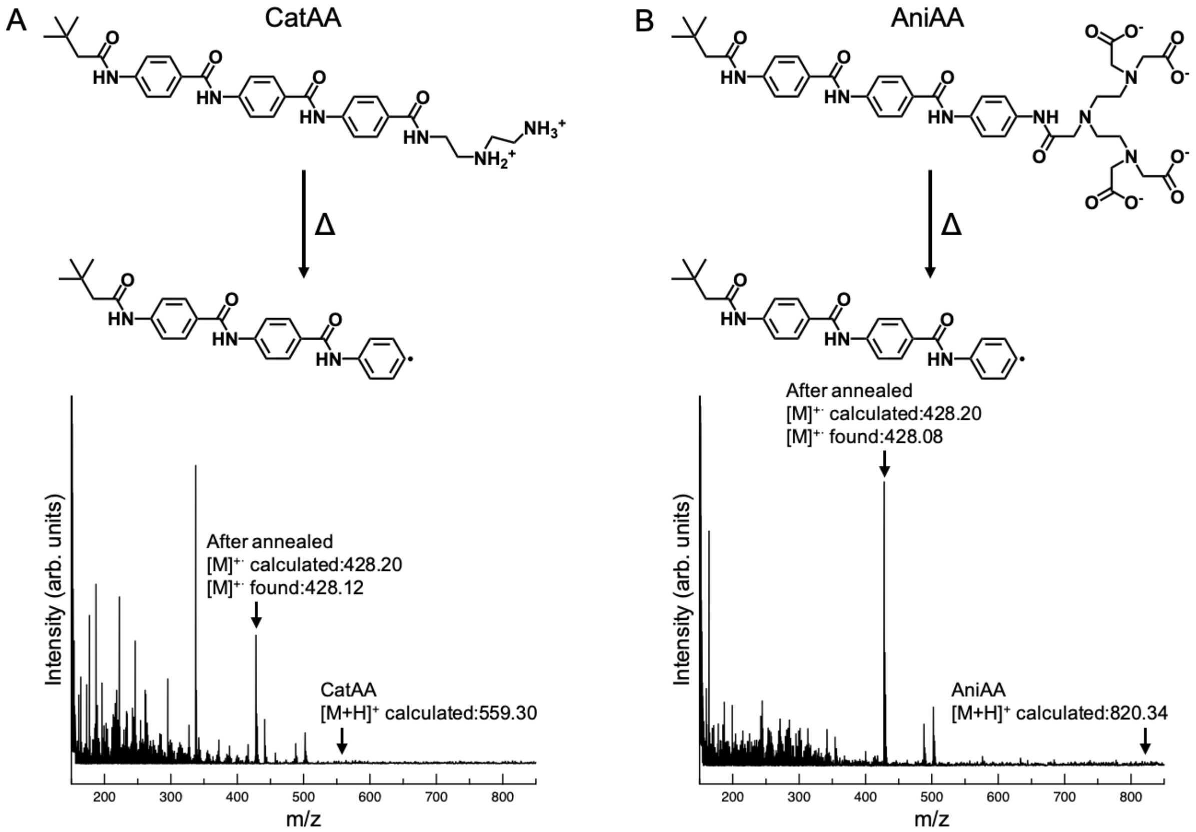

Supplementary Figure 15 | Chemical composition and corresponding direct analysis in real time (DART) mass spectra of annealed CatAA and AniAA. The radical terminated triaramid product is supported by ^{1}H NMR, ^{13}C NMR, FTIR, and DART mass spectrometry. The unassigned peaks do not correspond to any m/z values based on anticipated molecular structure fragments and are observed in instrument background runs without sample.

Supplementary Note 6: TEM of annealed nanoribbon

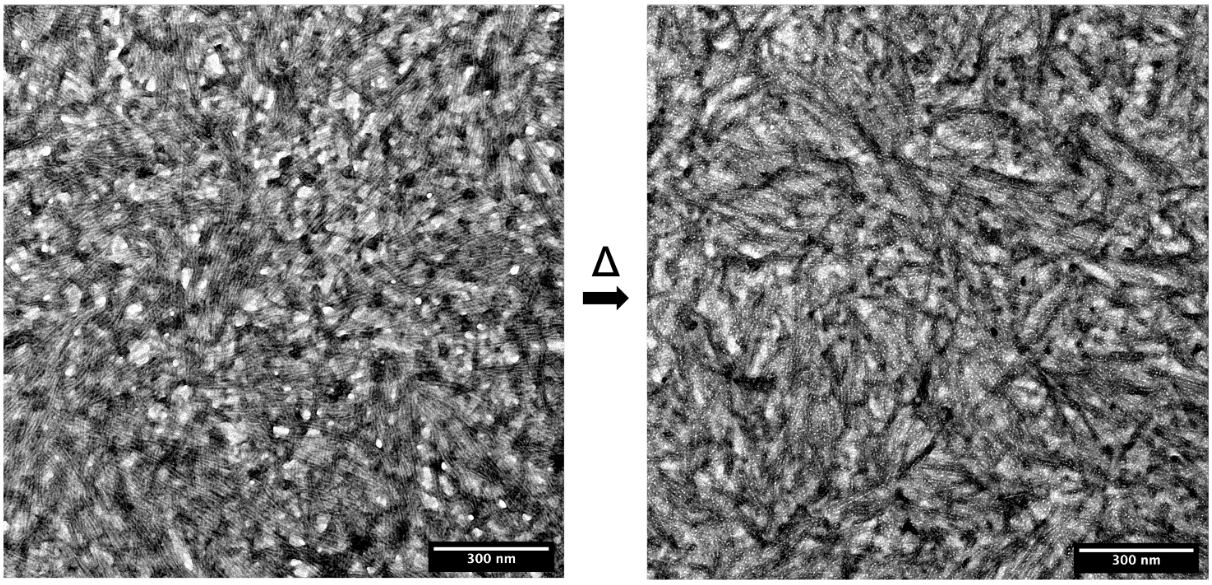

Supplementary Figure 16 | TEM of untreated CatAA nanoribbons before (left) and after annealing at 250°C for 1 h in air (right). The nanoribbon morphology is maintained after annealing.

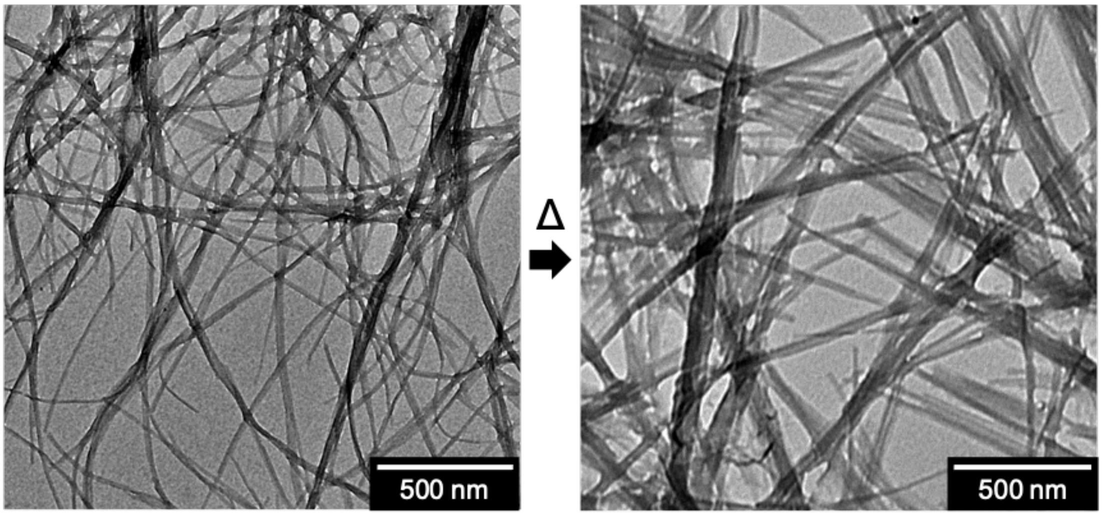

Supplementary Figure 17 | TEM of untreated AniAA nanoribbons before (left) and after annealing at 250°C for 1 h in air (right). The nanoribbon morphology is maintained after annealing.

Supplementary Note 7: Variable temperature WAXS

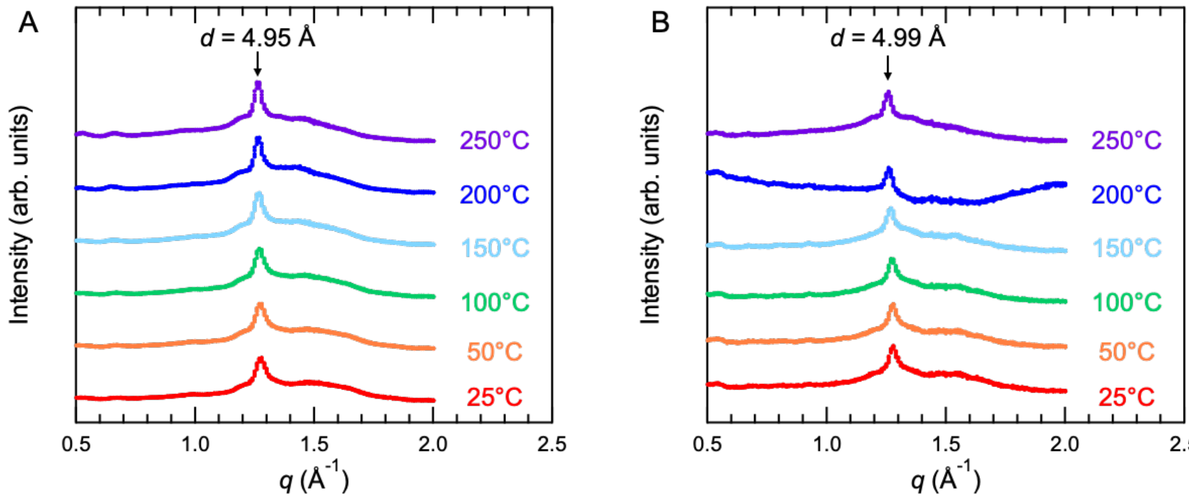

Supplementary Figure 18 | 1D WAXS profiles of lyophilized nanoribbons (**A**) CatAA and (**B**) AniAA annealed at different temperatures. The lyophilized nanoribbons are loaded into 2 mm I.D. quartz capillary tubes (Hampton Research). A Linkam HFSX-350 is used to heat the capillaries from rt to 250 °C. For each temperature point, samples are ramped to the desired temperature, thermalized for 5 min, and then analyzed to obtain the WAXS pattern with a 5 min exposure time. The WAXS profiles reveal the maintenance of molecular ordering through the heating process.

Supplementary Note 8: Disassembly of annealed nanoribbon

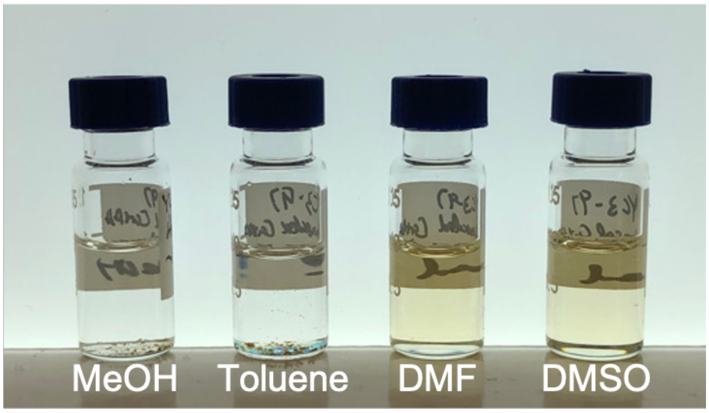

Supplementary Figure 19 | Samples prepared by dissolving annealed nanoribbons in several organic solvents. Methanol and toluene do not dissolve the nanoribbons, whereas aprotic solvents such as dimethylformamide (DMF) and dimethyl sulfoxide (DMSO) fully dissolve and disassemble the annealed nanoribbons. All samples are annealed CatAA nanoribbons equilibrated in solvent at 1 mg/mL for 24 h.

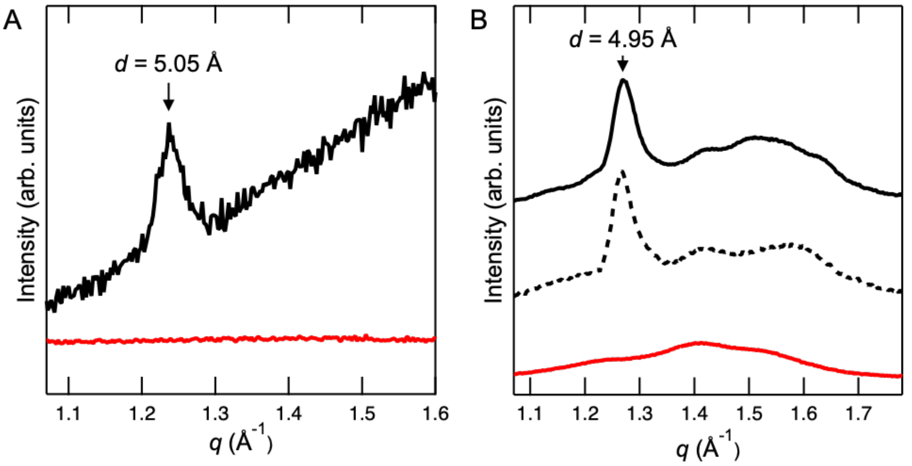

Supplementary Figure 20 | (**A**) 1D WAXS profiles of untreated CatAA nanoribbons in aqueous solution (black) and annealed nanoribbons resuspended in DMSO (red). The absence of the peak at *d* = 5.05 Å in DMSO confirms the full dissolution of the hydrophobic triaramid compound. (**B**) 1D powder WAXS profiles of untreated CatAA nanoribbons after lyophilization (black solid line), nanoribbons after annealing (black dotted line), and annealed nanoribbons after full disassembly in DMF, followed by drying (red). The absence of the peak at *d* = 4.95 Å, which corresponds to molecular packing in the hydrogen bonding direction, confirms that kinetically trapped nanostructures do not persist after exposure to DMF.

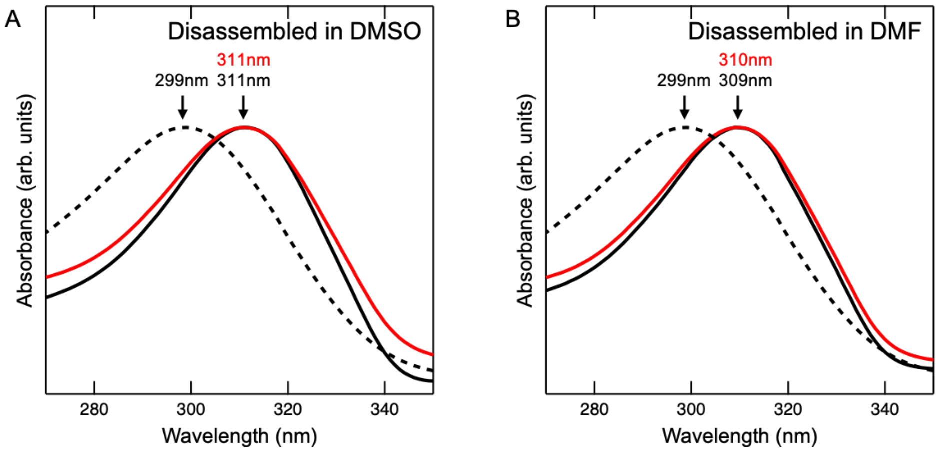

Supplementary Figure 21 | (A) UV-vis absorbance spectra of lyophilized untreated (black, solid) CatAA nanoribbons and annealed nanoribbons (red) resuspended in dimethyl sulfoxide (DMSO), with an aqueous solution of assembled CatAA nanoribbons (black, dotted) shown as a control. (**B**) UV-vis absorbance spectra of lyophilized untreated (black, solid) CatAA nanoribbons and annealed nanoribbons (red) resuspended in dimethylformamide (DMF) with an aqueous solution of CatAA nanoribbons (black, dotted) shown as a control. Red shifts of λ_{max} indicate dissociation of hydrogen bonding in the aramid domain occurs with dissolution of the CatAA and its annealed product in DMSO or DMF.

Supplementary Note 9: Solubility of decomposition product

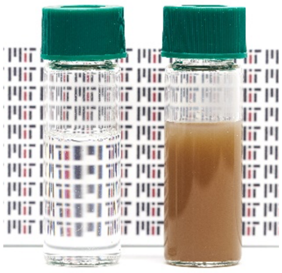

Supplementary Figure 22 | Lyophilized untreated (left) and annealed (right) CatAA nanoribbons resuspended in water. Changes in color and water solubility are consistent with the removal of the hydrophilic surface of CatAA nanoribbons after annealing.

Supplementary Note 10: WAXS of annealed nanoribbon threads

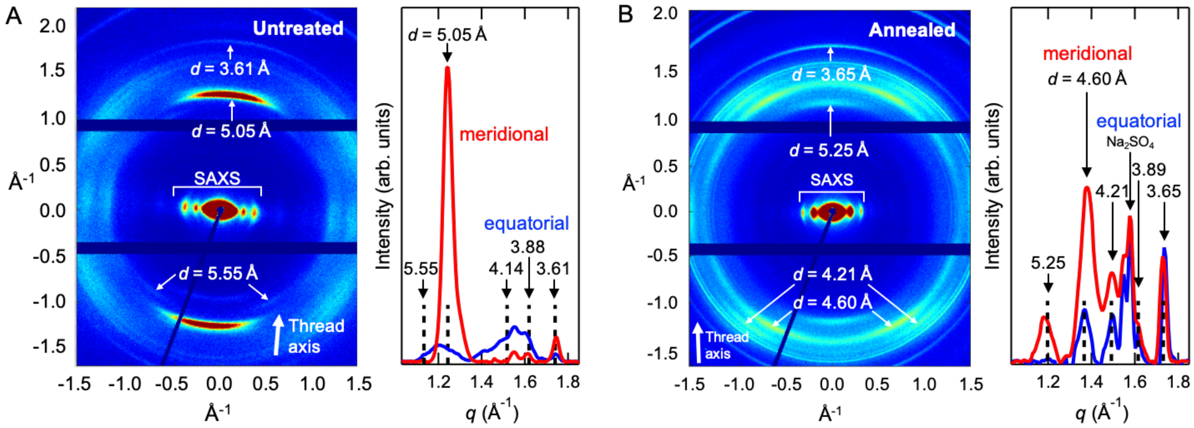

Supplementary Figure 23 | WAXS patterns and 1D scattering profiles of (**A**) untreated and (**B**) annealed AA nanoribbon threads. The 1D scattering profiles are obtained by azimuthal integration to capture meridional and equatorial scattering over 45° angles. Black dotted lines are simulated peak positions of the unit cell (space group 26:*Pmc*2$_1$) based on poly(*p*-benzamide), reported in the previous study[1]. The unit cell parameters of untreated AA nanoribbon thread are $a = 7.22$ Å, $b = 5.05$ Å and $c = 11.10$ Å, and the parameters of annealed AA nanoribbon thread are $a = 7.30$ Å, $b = 4.60$ Å and $c = 10.50$ Å. Isotropic peaks from Na_2SO_4 salt become more distinct with salt crystallization upon annealing.

Explanation of Supplementary Figure 23

The *d*-spacing corresponding to molecular packing in the hydrogen bonding direction decreases from 5.05 Å to 4.60 Å (a change of 9%) after annealing, whereas the *d*-spacing corresponding to the π–π stacking direction remains approximately the same upon annealing ($d = 3.61$ Å to $d = 3.65$ Å). We attribute this unit cell contraction along the hydrogen bonding direction to tighter spacings between molecules as intermolecular electrostatic repulsion decreases upon head group removal. These simulated results indicate that precise molecular organization is maintained in the solid-state upon head group decomposition.

WAXS patterns of the annealed thread show more complicated scattering patterns than the non-annealed thread likely due to the introduction of subtle disorder in the alignment of domains of bundled nanoribbons after heating. WAXS patterns of untreated threads show that there is one strong peak located on both sides of the meridional direction (5.05 Å) corresponding to the (010) plane of the fitted unit cell, and there is one subtle peak corresponding to the (002) plane. This (002) peak corresponds to $d = 5.55$ Å and is observed as four small arcs, one centered in each quadrant (corner) of the detector. Upon annealing, the (010) peak shifts from 5.05 Å to 4.60 Å and switches from the meridional direction to the 4 corner arcs with reduced relative intensity. Conversely, upon annealing, the (002) peak shifts from 5.55 Å to 5.25 Å and switches from the 4 corner arcs to the meridional direction with increased relative intensity. To summarize, the reversal of relative diffraction intensity and also of the positions of the peaks are likely caused by the introduction of subtle disorder in the alignment of domains of bundled nanoribbons after applied annealing.

Finally, we observe that the lattice parameters of the lyophilized powder (Fig. 3B) and untreated thread, or those of the annealed lyophilized powder (Fig. 3B) and annealed thread do not match

– the peaks do not shift before and after annealing for lyophilized powder. We believe this difference arises from 1) Na_2SO_4 salt in the thread altering the intermolecular electrostatic repulsion between head groups and therefore influencing the spacing of molecular packing, and 2) aligned nanoribbons in the thread potentially having residual stress from shear-alignment.

Supplementary References

1. Christoff-Tempesta T, *et. al.*, Self-assembly of aramid amphiphiles into ultra-stable nanoribbons and aligned nanofiber threads, *Nature Nanotechnology*, **16**, 447-454 (2021).

2. Mukherjee M, Kumar S, Bose S, Das CK. Kharitonov AP, Study on the Mechanical, Rheological, and Morphological Properties of Short Kevlar™ Fiber/s-PS Composites, *Polymer-Plastic Technology and Engineering*, **47**, 623-629 (2008).

3. Pyo D, Expert system approach for spectra-structure correlation for vapor-phase infrared spectra, *Vibrational Spectroscopy*, **5**, 263-273 (1993)

4. Coates J, Interpretation of Infrared Spectra, A Practical Approach. In *Encyclopedia of Analytical Chemistry*, John Wiley & Sons, Ltd, Chichester, UK, 1–23, (2008)

5. Smith BC, Organic Nitrogen Compounds V: Amine Salts, *Spectroscopy*, **34**, 30-37 (2019)

6. Jenjob R, *et. al.*, Enhanced conjugation stability and blood circulation time of macromolecular gadolinium-DTPA contrast agent, *Materials Science and Engineering: C*, **61**, 659-664 (2016).
